# Biocompatibility Study of a New Dental Cement Based on Hydroxyapatite and Calcium Silicates: Focus on Liver, Kidney, and Spleen Tissue Effects

**DOI:** 10.3390/ijms22115468

**Published:** 2021-05-22

**Authors:** Smiljana Paraš, Dijana Trišić, Olivera Mitrović Ajtić, Đorđe Antonijević, Božana Čolović, Damjana Drobne, Vukoman Jokanović

**Affiliations:** 1 Faculty of Science and Mathematics, University of Banja Luka, 51 000 Banja Luka, Republic of Srpska, Bosnia and Herzegovina; smiljana.paras@pmf.unibl.org; 2 Faculty of Dental Medicine, University of Belgrade, 11 000 Belgrade, Serbia; dijana.trisic@stomf.bg.ac.rs; 3 Institute for Medical Research, University of Belgrade, 11 000 Belgrade, Serbia; oliveram@imi.bg.ac.rs; 4 Vinča Institute of Nuclear Sciences, University of Belgrade, 11 000 Belgrade, Serbia; antonijevic@vin.bg.ac.rs (Đ.A.); bozana@vin.bg.ac.rs (B.Č.); 5 Institute of Anatomy, Faculty of Medicine, University of Belgrade, 11 000 Belgrade, Serbia; 6 Biotechnical Faculty, University of Ljubljana, 1 000 Ljubljana, Slovenia; damjana.drobne@bf.uni-lj.si; 7 ALBOS d.o.o., 11 000 Belgrade, Serbia

**Keywords:** biocompatibility, calcium silicates, dental cement, hydroxyapatite, subchronic toxicity

## Abstract

The effects of a new material based on hydroxyapatite and calcium silicates, named ALBO-MPCA, were investigated on the liver, kidney and spleen. The material was administrated orally for 120 days in an in vivo model in Wistar rats, and untreated animals served as a control. Hematological and biochemical blood parameters were analyzed. Qualitative histological analysis of tissues, change in mitotic activity of cells, and histological characteristics was conducted, as well as quantitative stereological analysis of parenchymal cells, blood sinusoids, and connective tissues. Additionally, the protein expressions of Ki67 and CD68 markers were evaluated. Histological analysis revealed no pathological changes after the tested period. It showed the preservation of the architecture of blood sinusoids and epithelial cells and the presence of mitosis. Additionally, the significantly increased number of the Ki67 in the presence of ALBO-MPCA confirmed the proliferative effect of the material noticed by stereological analysis, while immunoreactive CD68 positive cells did not differ between groups. The study showed non-toxicity of the tested material based on the effects on the hematological, biochemical, and observed histological parameters; in addition, it showed evidence of its biocompatibility. These results could be the basis for further steps toward the application of tested materials in endodontics.

## 1. Introduction

Calcium silicate cement has long been used in endodontics, for various endodontic indications, such as pulp capping, pulpotomy, apexogenesis, root-end filling, etc. [1,2,3,4]. The mineral trioxide aggregate (MTA) is certainly one of the most widely investigated calcium silicate-based materials due to its biocompatibility, bioactivity, and hard-tissue conductive and inductive properties [5,6,7,8,9]. 

Despite many good properties, MTA shows many drawbacks during clinical application, such as long setting time, difficult handling, and potential teeth discoloration [10,11,12]. Therefore, researchers in this field resulted in a synthesis of new materials that could overcome the limitations of MTA [13].

In such an attempt, a new material based on hydroxyapatite and calcium silicates (named ALBO-MPCA) has been synthesized recently [14]. It combines the advantages of hydroxyapatite with excellent biocompatibility and bioactivity along with similarity with inorganic phases of biomineralized tissues. It has also the advantages of calcium silicates whose progressive hydration enables the transition of a hydrating cement paste from a fluid to a rigid material as a continuous process. Many biocompatibility aspects of tested cement have been already investigated at in vitro and in vivo conditions and compared to those of MTA [14,15,16,17,18,19,20], but their biocompatibility has to be further analyzed to assure absolute safety of application of this new material. 

Most of the studies dealing with the biocompatibility of the calcium silicates are in vitro studies. The in vitro studies provide a basic understanding of material–cell interactions, but long-term adverse effects could not be predicted. Additionally, calcium silicate materials often contain impurities that can induce a toxic systemic effect. For instance, the first formulations of MTA were not fabricated from laboratory manufactured calcium silicates without impurities, but Portland cement was used instead. Thus, both MTA and Portland showed evidence of heavy metals (arsenic, lead, and chromium) in the acid-soluble form and they are proven to release more arsenic than the amount specified in ISO 9917-1 [21]. In addition, it has been demonstrated that ProRoot MTA consists of heavy metals such as copper, manganese, iron, strontium [22]. It is also noteworthy that MTA Angelus, MTA Fillapex, or Theracal LC affects the rats’ brain aluminum levels and oxidative stress parameters [23]. Consequently, the in vivo testing of material is unavoidable when a material is intended for oral application.

Qualitative histological analysis was supported by further quantitative, stereological analysis, providing more objective and precise analysis [24,25]. Furthermore, immunohistochemical analysis of tissue sections provided better insight into histological status of treated and control animals. In our previous study, we standardized the use of these two markers to test the toxicity and biocompatibility of the tested nanomaterials [26]. The most commonly used proliferation-associated marker Ki-67 is a nuclear antigen present only in proliferating cells [27]. Bearing in mind that Ki67 is a marker of proliferation, we used it in our study to prove that the experimental tissue cells were not damaged and did not undergo higher level of apoptosis compared to the control group of animals. Macrophages are required during kidney development and appear in the initiation and propagation of renal injury. The obtained findings provide valuable information on the participation of macrophages in the regeneration of rat kidney. This information may be useful for evaluation of renal toxicity when macrophages are involved in the development of renal injury [28]. According to this, we used CD68 markers in our study.

The work presented here aimed to examine the biocompatibility of new material based on hydroxyapatite and calcium silicates (named ALBO-MPCA) in vivo. The material was administered orally to Wistar rats daily for 120 days, and the systemic toxicity was examined by hematological and biochemical blood parameters, histological parameters of the liver, kidneys, and spleen, and two selected molecular markers (Ki67 and CD68).

## 2. Results 

### 2.1. Health Status of the Rats during the Experimental Period

**Figure 1:**
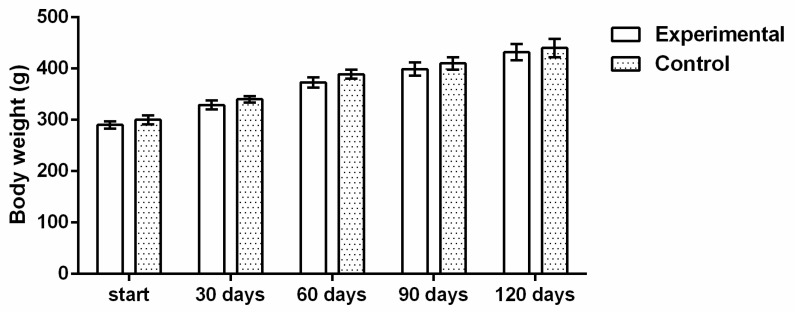
Average body weight after treatment (*n* = 12) and control (*n* = 9) during the study.

No changes in usual behavior of the rats, as well as changes in skin and hair, food and water consummation, or urinating and defecation, were noticed during daily observation of the rats during the experiment. Mildly and consistently increased body weight was observed through the experiment (Figure 1).

### 2.2. Blood Parameters

Blood analysis revealed that the values of the analyzed blood parameters (hemoglobin, leukocytes, platelets, ALT, AST, ALP, urea, creatinine, bilirubin) did not significantly differ between the control and treated group (Table 1).

### 2.3. Histological and Stereological Parameters

#### 2.3.1. Histological and Stereological Parameters of the Liver Tissue

**Figure 2:**
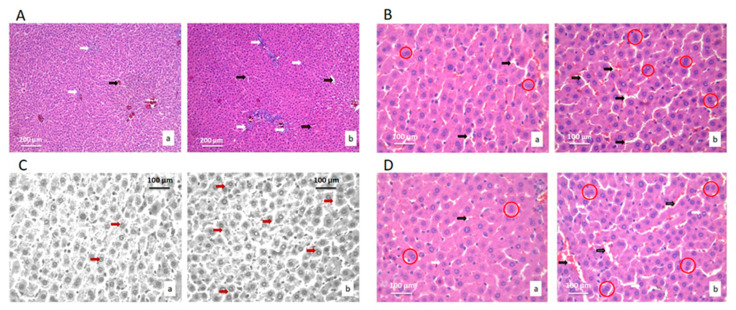
Micrographs of the histological cross-section of the liver of the control group (a) and treated group (b), (H&E). (**A**) White arrows show connective tissue and black show blood vessels. Magnification 20×. (**B**) Black arrows show hepatocytes nuclei, and red circles show hepatocytes with two nuclei. Magnification 50×. (**C**) Red arrows show hepatocytes nuclei. Magnification 50×, digitally processed RGB technique. (**D**) Black arrows show capillary sinusoids, and red circles show hepatocytes with two nuclei. Magnification 50×.

Histological and stereological analyses of liver tissue of treated and control rats were based on the comparison of fifteen stereological parameters (Table 2). Histological analysis showed the preservation of the hepatocytes and blood sinusoids architecture in livers of treated animals. No pathological changes were observed in the liver tissue of treated rats, such as cysts, fibrosis, loss of hepatocytes, necrosis, or lymphocyte agglomerates due to inflammatory reactions (Figure 2).

The stereological parameter of volume density of capillary sinusoids increased (*p* < 0.05) in the liver tissues of treated rats in comparison to the control group. Additionally, the number, numerical density and nucleocytoplasmic ratio (NCO) of hepatocytes all increased in the experimental group compared to the control (*p* < 0.05) (Table 2). 

The liver tissue images showed an increase in the number, numerical density and hepatocytes in active mitosis in the treated group. Additionally, in the treated group, there was a higher number of hepatocytes with two nuclei. 

Stereological analysis of the liver tissue also revealed the increase in the number and numerical density of the connective tissue cells in treated group (*p* < 0.05). The numerical values of the stereological parameters of blood sinusoids showed an increase in all of the compared parameters in the treated rats compared to the control (*p* < 0.05) (Table 2).

#### 2.3.2. Histological and Stereological Parameters of the Kidney Tissue

**Figure 3:**
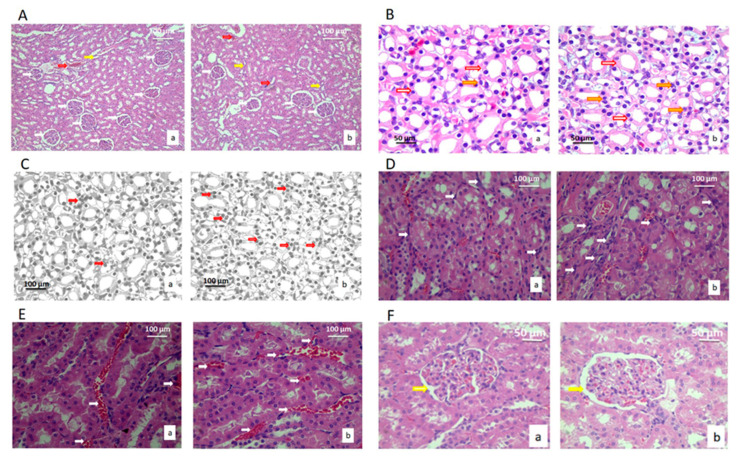
Micrographs of histological cross-sections of the kidney of the control group (a) and treated group (b), (H&E). (**A**) White arrows show glomeruli, red arrows show blood sinusoids, and yellow arrows show connective tissue. Magnification 20×. (**B**) Yellow arrows show nuclei, and red arrows show epithelial cells of collecting ducts. Magnification 50×. (**C**) Red arrows show the epithelial cells of collecting ducts. Magnification 50×; digitally processed RGB technique. (**D**) White arrows show the connective tissue of collecting ducts. Magnification 50×. (**E**) White arrows show the blood sinusoids of the collection ducts. Magnification 100×. (**F**) White arrows show the distance between glomeruli and Bowman’s capsule. Magnification 100×.

There were no pathological changes in renal tissue in the treated rats, such as cysts, fibrosis, and loss of collecting duct cells, loss of glomeruli, necrosis, or lymphocyte agglomerates due to inflammatory reactions (Figure 3). Histological and cytological analysis of the kidneys was based on the comparison of seventeen stereological parameters (Table 3). All parameters determine the morphological changes that could affect the function. 

The volume densities of the blood sinusoids were higher in the treated group of animals compared to the control (*p* < 0.05). Additionally, the number of the epithelial cells in blood sinusoids between the collecting ducts of the kidney and NCO was significantly higher in the treated group, as well as capillary endothelial cells (*p* < 0.05) (Table 3). 

#### 2.3.3. Histological and Stereological Parameters of the Spleen Tissue

**Figure 4:**
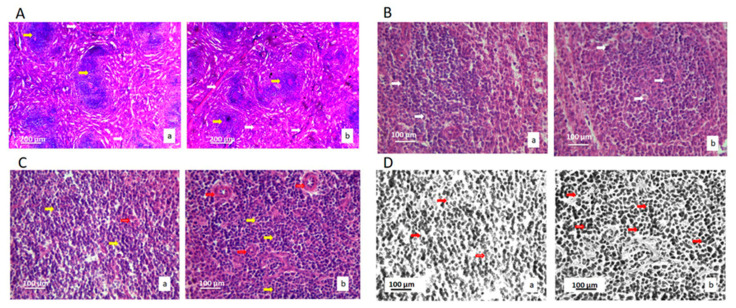
Micrographs of the histological cross-section of the rat spleen of the control group (a) and the treated group (b), (H&E). (**A**) White arrows show connective tissue, and red arrows show lymphatic tissue. Magnification 200×. (**B**) White arrows show epithelial cells. Magnification 50×. (**C**) Yellow arrows show lymphocytes, and red arrows show blood vessels. Magnification 50×. (**D**) Red arrows show lymphocytes. Magnification 50×; digitally processed RGB technique.

Histological and cytological analyses of the spleen of the treated and the control rats were based on the comparison of eighteen stereological parameters (Table 4). The analyzed parameters could indicate changes in the spleen tissue, epithelial cells, lymphocytes, connective tissue and capillary sinusoids. There were no pathological changes in the tissue of the spleen, such as micro lesions, a change in the architecture of histology of the spleen, a large loss of lymphocytes, epithelial or star cells, fibrosis, necrosis, or large lymphocytes accumulation due to inflammatory reactions (Figure 4). 

Volume density of lymphocytes and blood capillaries increased in the treated group (*p* < 0.05) (Table 4). The lymphocytes increased in their number by mean value of 24.71% and this increase was significant (*p* < 0.05), as well as its numerical density. A large number of mature lymphocytes can be seen in the tissue of the spleen, in the sinusoids, and between the star’s epithelial cells. Lymphocytes were more densely colored and evenly distributed. The connective tissue of the spleen was not altered when the treated and control animals were compared.

### 2.4. Expression of Proteins Ki67 and CD68 in Liver, Kidney, and Spleen

**Figure 5:**
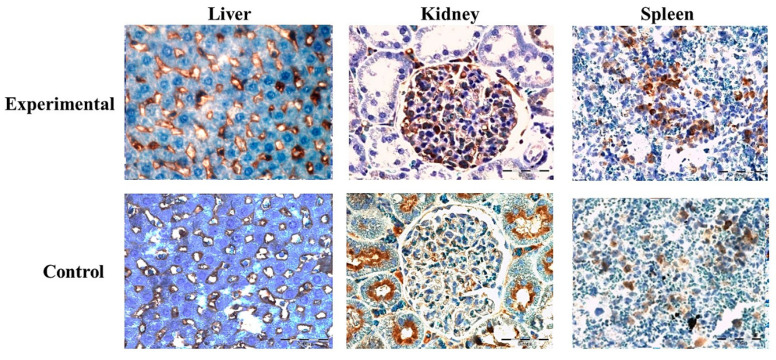
Representative images of Ki67 protein expression in rats’ liver, kidney and spleen tissue sections of experimental and control group, 40× magnification. A significant expression of Ki67+ cells was observed in experimental group (brown colored cells).

**Figure 6:**
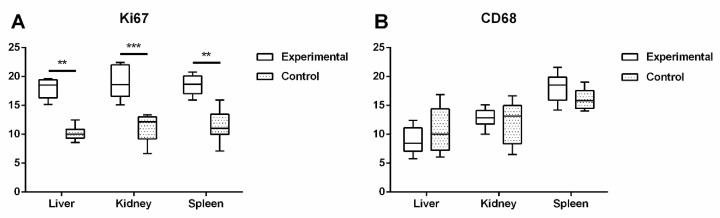
Percentage area of Ki67 (**A**) and immunoreactive CD68+ (**B**) stained cells was calculated with ImageJ using 3 images per organs (*t*-test, ** *p* < 0.01, *** *p* < 0.001).

Expression of Ki67 protein was significantly higher in the experimental group of liver (*p* < 0.01), spleen (*p* < 0.003), and kidney tissue samples (*p* < 0.001), in comparison to the control (Figure 5 and Figure 6A). 

**Figure 7:**
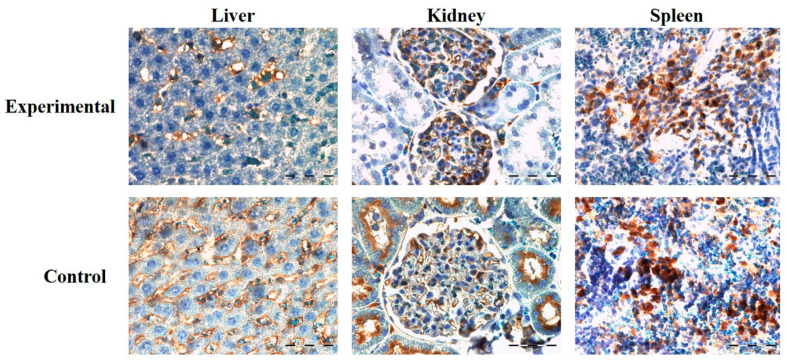
Representative images of CD68 protein expression in rats’ liver, kidney, and spleen tissue sections of experimental and control group, 40× magnification. Mild expression of CD68+ immunoreactive cells was observed in all groups (brown colored cells).

Protein expression of CD68 immunoreactive cells in kidney and liver was higher in the control in comparison to the experimental group, with no significant difference. In the spleen tissue sections, CD68-specific protein expression was higher in the experimental group but also showed no significant difference in comparison to the control (Figure 6B and Figure 7). 

## 3. Discussion

In this study, ALBO-MPCA biocompatibility was investigated over the subchronic 120 days’ oral exposure study using Wistar rats. Organ toxicity was investigated by analyzing liver, kidneys, and spleen histopathological changes, as well as by hematological and biochemical parameters. The present study was conducted in 21 animals (12 in the experimental group and 9 in the control group) to prove the concept. All experiments were conducted following the guiding principles of animal welfare, of so-called three Rs, refinement, reduction and replacement. This means that every effort must be made: to refine the procedures so that the degree of suffering is kept to a minimum, to reduce the number of animals used in research to the minimum required for meaningful results, and to replace the use of live animals by non-animal alternative. Bearing all this in mind, the concept was investigated on 21 animals to avoid sacrificing a huge number of animals. In addition, the significance of the obtained results was strengthened by detailed analysis of different segments of liver, kidney and spleen histology. A similar principle was used in previous papers [26,29].

The gross morphology of the liver of the treated rats was normal, although there were statistically significant changes in the parameters of endothelial cells of the blood sinusoids. Hepatocytes and hepatic plaques were normal and neatly placed, without abnormalities around the central vein. The same results on the biocompatibility of bio-Mg-Zn material examined on changes in liver tissue were published with the assertion that there was no change in the architecture of hepatocytes and the morphology of the liver lobules [30]. 

Stereological parameters allow for quantitative detection of possible effects of tested material on rat liver tissue. As presented, more of the liver cells in the treated rats were in active mitosis than those from the control group. The increased mitotic index and the number of hepatocytes with two nuclei could be explained by the liver response to ingested foreign material [13]. Additionally, hepatocytes nuclei surface areas and NCO increased, while whole cell surface areas decreased, which also suggests proliferation of hepatocytes as a sign of adaptation and intensified liver function. Since there were no other adverse effects observed by using other parameters, we suggest that the increased NCO ratio is not indicative of a pathological outcome of exposure but rather a physiological response to intake of material. Namely, the changes in the cytoplasmic volume can occur due to several factors, including osmotic stress and response to different cellular signals [31].

As a consequence, the parameters of the blood sinusoids and the number of endothelial cells also increased following the change in the number of hepatocytes. The statistically significant increase in the number and density of capillary endothelial cells indicated increased blood flow through the liver in experimental group, compared with control group, possibly due to more intense blood filtration. Consequently, the significant increase in the number of endothelial cells (*p* < 0.05) due to the formation of new blood sinusoids evidenced the process of the necessary adaptation of the rats’ livers to the tested material. Depending on the Ca^2+^ ion concentration in cells and the interaction with lipid-soluble ionophores, Ca^2+^ ion transporters could induce increased transport Ca^2+^ ions in liver sinusoidal endothelial cells, their contraction and consequently diminished blood flow. Therefore, an increased number of balanced endothelial cells induced blood flow through sinusoids and the formation of the new blood sinusoids, showing adequate liver tissue reaction on the material [13,30,32]. 

Furthermore, the number and numerical density of the connective tissue cells in liver significantly increased in the experimental group, while no differences in structure or density were observed after 4 months of exposure to the tested material. Similar to the results presented here, in previous studies of systematic toxicity of new endodontic materials, increased mitotic activity of parenchymal cells was accompanied by the increased density of the stromal cells [19,33]. Immunostaining showed significant increase in Ki67 protein in the livers of the treated animals, supporting the findings of parenchymal proliferation [34] but without differences in the expression of CD68 protein, which indicated that no macrophage proliferation occurred in response to subchronic exposure to the tested material [35]. 

The morphology of glomeruli and the tubes in the kidneys of the treated rats were not affected and without signs of inflammation after exposure period. The absence of any morphological changes in the kidney tissue was earlier reported in the study with implantation of biocompatible Mg-Nd-Zn-Zr material into the bone of the rabbit, which suggested that the tissue of the kidneys remained normal after the animal carried the implant for experimental period [36]. 

Stereological analysis of blood sinusoids between the collecting ducts of the kidney indicated a statistically significant increase in the number of capillary endothelial cells (*p* < 0.05), similar to the previously described increase in parameters of blood sinusoids and number of endothelial cells in liver. Due to increased blood circulation, the volume of the epithelial cells increased. Similar to our results, the study of the mineral trioxide aggregate effect on the liver and kidney function reported that kidney function subsequently increased after a month of MTA treatment, while the liver function increased earlier, during the first week, and further increased to the end of the experiment [13].

The spleen of rats fed with extract of the tested material retained a healthy architecture, while the lymphocytes resemble the position and density of these in control animals. The only significant difference detected when compared to the control animals and exposed ones was the induced number of lymphocytes in animals fed for 4 months with material. In the previous report, the spleen showed the greatest tolerance and lack of response to the implantation of biodegradable magnesium implants, where its tissue remained unchanged and normal after four weeks of the experimental period [37]. 

Furthermore, as it is well known that spleen important function is to filter blood-borne pathogens and antigens, an increased number of lymphocytes in the spleen could be explained by higher blood perfusion and, thus, the presence of a high concentration of the materials’ extract in organism during 4 months of investigations [38]. As hematological analysis revealed no increase in lymphocytes in systematic circulation in the experimental group compared to the control, it implies the preserved physiological nature and reaction of spleen cells to the subchronic presence of materials based on calcium in the organism.

Immunostaining confirmed parenchymal proliferation in the spleen by the increased expression value of the Ki67 protein in the experimental group, while the number of immunoreactive CD68 positive cells remained in physiological range. As a protein involved in the regulation of cell cycle as DNA replication, and higher-order chromatin organization, its higher expression is confirmed also by histological analysis, indicating a higher number of mitosis and, thus, a higher number of cells in the presence of material extract [39,40]. Ahmed and sar. (2017) also investigate the effect of silver nanoparticles on testes of adult albino rats (histological, immunohistochemical and biochemical study), and Ki67 and CD68 markers were used in the study. Results of their study showed a significant decrease in cell proliferation and an increase in interstitial tissue macrophages in the experimental groups in comparison to their control group [41]. Additionally, in our previous study, the effect of calcium silicate and calcium aluminate implantation on systematic subchronic toxicity was evaluated. The results showed that using materials induced normal and reversible response in the liver. Namely, investigated proliferation rate with the average number of immunoreactive Ki67 positive parenchymal cells was also reported, with no visible signs of pathological changes or immunological reaction to the materials [42].

As investigated previously, the blood volume in the rats is 4.7–8.0 mL/100 g body volume (BW) (mean value: 7.0 mL) [43]. As in our study, BW was between 260 and 280 g, and following Lee and Blaufox’s research [43], the blood quantity per animal was approximately 18.2 to 19.6 mL. Experimental animals were fed with 1 mL/day for 120 consecutive days with saturated solution (100 mg/mL), which makes 120 mL of the total quantity of used extract during experiment, a 6.1–6.6 times higher volume in comparison to the animals’ total blood volume. 

It can be concluded that even though animals were exposed to the exceptional high Ca^2+^ ion concentration in regard to the blood volume, the histological analysis of the livers, kidneys, and spleens did not detect significant changes in tissue morphology, suggesting that the used dental cement is biocompatible.

## 4. Materials and Methods

### 4.1. Test Material

The test material (ALBO-MPCA) consists of 40% hydroxyapatite, 20% calcium silicates, 20% gypsum, and 20% BaSO_4_ as a radiopaque agent. Synthesis methods of hydroxyapatite and calcium silicates were described in detail in our previous investigations [14,15,16], while gypsum dehydrate and BaSO_4_ were purchased from Whip Mix Corporation, USA, and Merck, Germany, respectively. Brief structural and morphological analysis of the material is given in the Supplementary Materials, while detailed physicochemical characterization can be found in our previous investigations [14,15,16].

For the purpose of this study, aqueous extract of the material was used, obtained by immersion in distilled water in concentration of 100 mg/mL and decantation after 5 days (according to the standard ISO 10993-12:2012—Biological Evaluation of Medical Devices– Part 12: Sample Preparation and Reference Materials).

### 4.2. Experiment Design

Experimental protocol for this study was approved by the Ethical Committee of the Faculty of Veterinary Medicine, Belgrade, and Ministry of Agriculture and Environment Protection of the Republic of Serbia (decision number 01-831/2) and realized under recommendations of the European Communities Council Directive (86/609/EEC). 

Male Wistar rats, 8–12 weeks old, with average weight of 260–280 g, were used as animal models. The rats were placed in Plexiglas cages, one animal per cage, and had access to food and water ab libitum. The air temperature in the vivarium was 23 ± 3 °C, while the humidity was 55 ± 5%, with a 12/12 light–dark cycle.

The rats were randomly divided into two experimental groups: treated group (*n* = 12) and control group (*n* = 9). The animals in the treated group received orally 1 mL of the aqueous extract of the tested material (≈400 mg/kg body wt) daily for 120 days, and the animals in control group received 1 mL of distilled water, all by using a syringe feeding technique. Health status of the experimental rats, including behavior, changes in the skin and hair, food and water consummation, urinating, and defecation, was checked daily. Body weight was measured at the beginning of the experiment and 4 times during the experiment.

On the last day of the experiment, blood samples were taken from all the rats for analysis of hematological (hemoglobin, leukocytes, and platelets) and biochemical parameters (alanine aminotransaminase (ALT), aspartate aminotransaminase (AST), alkaline phosphatase (ALP), urea, creatinine, and bilirubin).

After blood sampling, the rats were sacrificed by intravenous administration of thiopentone sodium solution in a dose of 170 mg/kg. The rats’ livers, kidneys, and spleens were removed and immersed in Buoin’s fixative solution for 24 h, cut in pieces for better penetration of solutions, and immersed into fresh Buoin’s solution. Dissected pieces of organs were processed following standard tissue preparation procedures for histological sample preparation. Chemically preserved tissue was embedded in paraffin, followed by sectioning, and staining with hematoxylin–eosin (H&E) (Merck, Darmstadt, Germany). Tissue sections of 4 µm thickness were cut using rotary microtome (Leica rotary Microtome RM 2165, Leica Microsystems, Wetzlar, Germany), and every 5th section was used for the microscopic observation. The qualitative analysis of the microscopic slides was performed using the light microscope Leica DM8000 M with MEGA VIEW camera and software system for digital image transfer. 

### 4.3. Histological and Stereological Analysis of the Liver, Kidney, and Spleen Tissue

Histological and stereological unbiased analysis assumed determination of stereological parameters on the stained cross-sections of livers, kidneys, and spleens of the treated and control rats. Stereological measurements were performed using a stereological universal test system based on Cavalier’s principle, using a 16.0 point-counting system MBF software system Application Suite 3.0.0. (MBF Bioscience, Williston, VT, USA), with a P2 spacing grid with the maximum magnification of microscope x400. Micrographs were acquired in RGB layout and converted to binary format for stereological analysis [24,25].

The analyzed stereological parameters of the liver tissue were: volume density, number, surface area, and numerical density of hepatocytes, blood sinusoids, and connective tissue, as well as the surface area of the nuclei, the nuclear receptor coactivator (NCO) and the mitotic index of hepatocytes and endothelial cells of liver parenchyma. 

The analyzed stereological parameters of the kidney tissue were: volume density, number, surface area, and numerical density of epithelial cells of collecting canals, endothelial cells of capillaries and connective tissue cells, as well as volume density, surface area and spacing of the glomeruli and Bowman’s capsule, and the surface area of the nuclei and the NCO of epithelial cells of the collecting ducts. 

The analyzed stereological parameters of the spleen tissue were: volume density, number, surface area, and numerical density of epithelial cells, lymphocytes, connective tissue cells and endothelial cells of the blood capillaries, as well as the surface area of the nuclei and NCO of epithelial cells.

Tissue sections of rats’ livers, kidneys, and spleens, stained with H&E, were used for measuring the numerical density (Nv) and the number of all epithelial cells per unit volume of cells and sinusoids. Volume density (Vv) of all epithelial cells and sinusoids was calculated using the formula: Vv = Pf/Pt (mm^0^), where Pf is the number of matches on the test system of the desired phase (all epithelial cells or lumen of sinusoids) and Pt is the total number of points of the test system [44]. 

Liver, kidney, and spleen tissue micrographs were acquired in RGB format and converted to binary format for analyzing the number and numerical density of their endothelial cells. The number of matches on the test system was determined using the following principle: all nuclei of endothelial cells and lumen of sinusoids were marked as reference point, while the cell surface areas were calculated assuming that the cells have visible contours and nuclei within the test system, the cell surface areas and their contours do not touch the frame of the test system, and the cells’ contours are visible in the test system without a visible nucleus. Numerical density (Nv) of all epithelial cells and sinusoids was calculated based on the cell count (*Q*) in the volume of the analyzed tissues (*Vo*):

$$\mathrm{Nv}\;=\;\frac{Q}{Vo}\;=\;\frac{Q}{\sum_{i=1}^{n}Pi\times a\times h}\;({\mathrm{mm}}^{-3})$$

where Σ*Pi* is the number of counted frames, *a* is the space of counted frame (*a* = 25,002), and *h* is the height factor, which is equal to the width of the histological section (4 μm) [45].

The values of the surface areas and volumes of all epithelial cells, as well as the surface areas and volumes of their nuclei, were determined using the values of their diameters measured with the MBF software system. Nucleocytoplasmic ratio (NCO) was determined as the quotient between the volume of nuclei of endothelial cells and volume of their cytoplasm. Mitotic index of hepatocytes was estimated by counting hepatocytes in optical fields on each slide with maximum microscope magnification of ×200. The mitotic index was determined as the ratio between the number of hepatocytes in mitosis and the total number of hepatocytes in ten visible fields per slide.

### 4.4. Immunohistochemical Analysis

For immunohistochemical analysis, tissue sections of 5 µm thickness were heated at 56 °C for 60 min and through the series of xylenes and alcohols deparaffinized and rehydrated. To block endogenous peroxidase activity, tissue sections were treated with 3% H_2_O_2_ solution in PBS. Furthermore, for epitope retrieval, tissue sections were heated in 10 mmol/L citrate buffer pH 6.0, in microwave oven, at 680 W for 21 min. Tissue sections were incubated with antibodies (against CD68 (RTU-CD68, Leica, LOT 6000698) and Ki67 protein (Monoclonal Mouse Anti-human Ki67 Antigen, Clone Ki-S5 (dilution 1:100)) overnight, in the humid chamber, at +4 °C. Streptavidin–biotin technique was used for immunostaining (LSAB+/HRP Kit, Peroxidase Labeling, K0690, DAKO Cytomation, Denmark). Immunoreactivity complex was visualized with DAKO Liquid DAB+ Substrate/Chromogen System (Code No. K3468) and counterstained with Mayer’s hematoxylin (Merck). For a negative control, the tissue sections with omitted primary antibody were used. As a positive control, the tissue sections known to express CD68 and Ki67 were used. Tissues were analyzed by a light microscope (Olympus AX70, Hamburg, Germany) with 40× magnification. In all tissue sections (experimental group—12 animals; control group—9 animals), five hot spots were used for quantification of Ki67/CD68 expression. Image analysis was performed in ImageJ (The National Institutes of Health, Bethesda, MD, USA). Ki67/CD68 were quantified by determining the positive expressed Ki67/CD68 areas (brown-colored cells) in microscopic fields, based on the threshold [46]. Median values of CD68 and Ki67 immunostaining were calculated for each tissue sample and used for further analysis.

### 4.5. Statistical Analysis

All values of the performed measurements were presented as mean values and standard deviations. Group comparisons were performed using parametric (*t*-test) and nonparametric test (Mann–Whitney U-test) depending on data distribution. All *p* values less than 0.05 were considered significant. Statistical software SPSS 20.0 (IBM Corp., Armonk, NY, USA) was used for data processing.

## 5. Conclusions

Histological and stereological analyses of the liver, kidney, and spleen tissue showed that daily oral administration of material extract through a prolonged period (120 days) induced no observed adverse histopathological response. Significant increase in the average number of Ki67, while showing no difference in the number of tissue immunoreactive CD68-positive cells in the presence of material, evidences cell proliferation in parenchymal and stromal tissue as a reaction to material presence, while no immunological reaction occurred. This study suggests good biocompatibility of novel nanostructured endodontic material and marked it as an excellent candidate for future investigations.

## Figures and Tables

**Table 1:** Results of blood analysis; *t*-test, *p* < 0.05.

	Experimental (*n* = 12)	Control (*n* = 9)
Hemoglobin	137 ± 8	114 ± 6
Leukocytes	5.3 ± 0.9	6.2 ± 0.9
Platelets	520 ± 170	630 ± 150
ALT	70 ± 30	60 ± 30
AST	600 ± 600	700 ± 700
ALP	63 ± 8	69 ± 8
Urea	5.1 ± 0.8	5.2 ± 0.5
Creatinine	38 ± 5	40 ± 5
Bilirubin	1 ± 1	1.3 ± 0.5

**Table 2:** Stereological parameters of the liver of the control and treated groups of rats; values are shown as mean ± SD; Mann–Whitney U-test, * *p* < 0.05.

Parameter	Experimental (*n* = 12)	Control (*n* = 9)
Volume density of hepatocytes (mm^0^)	0.69 ± 0.04	0.66 ± 0.05
Volume density of capillary sinusoids (mm^0^)	0.22 ± 0.02 *	0.16 ± 0.01
Volume density of connective tissue (mm^0^)	0.14 ± 0.010	0.13 ± 0.003
Number of hepatocytes	290,000 ± 30,000 *	255,168 ± 21,823
Numerical density of hepatocytes (mm^−3^)	49,600 ± 1100 *	44,575 ± 2633
Surface area of hepatocytes (μm^2^)	148 ± 9	153 ± 7
Surface area of hepatocytes nuclei (μm^2^)	50 ± 4	47 ± 2
NCO of hepatocytes	0.400 ± 0.02 *	0.324 ± 0.023
Mitotic index of hepatocytes	1.75 ± 0.03	1.6 ± 0.2
Number of connective tissue cells	135,000 ± 17,000 *	127,486 ± 17,518
Numerical density of connective tissue cells (mm^−3^)	26,000 ± 3000 *	21,934 ± 2367
Surface area of connective tissue cells (μm^2^)	101 ± 3	100 ± 3
Number of capillary endothelial cells	318,007 ± 13,600 *	275,916 ± 12,907
Numerical density of capillary endothelial cells (mm^−3^)	53,000 ± 3000 *	48,069 ± 2807
Surface area of capillary endothelial cells (μm^2^)	83 ± 3 *	75 ± 4

**Table 3:** Stereological parameters of kidneys of the control group and treated group of rats; values are shown as mean ± SD; Mann–Whitney U-test, * *p* < 0.05.

Parameter	Experimental (*n* = 12)	Control (*n* = 9)
Volume density of epithelial cells of collecting canals (mm^0^)	0.36 ± 0.03	0.33 ± 0.04
Volume density of blood sinusoids (mm^0^)	0.28 ± 0.04 *	0.22 ± 0.03
Volume density of connective tissue (mm^0^)	0.12 ± 0.01	0.10 ± 0.01
Volume density of glomeruli (mm^0^)	0.28 ± 0.03	0.30 ±0.04
Number of epithelial cells of collecting ducts	143,497 ± 1278 *	138,147 ± 3332
Numerical density of epithelial cells of collecting ducts (mm^−3^)	21,004 ± 1950	19,972 ± 2621
Surface area of epithelial cells of collecting ducts (μm^2^)	218 ± 29	204 ± 50
Surface area of nuclei of epithelial cells of collecting ducts (μm^2^)	64 ± 2	63 ± 3
NCO of epithelial cells of collecting ducts	0.30 ± 0.02 *	0.26 ± 0.03
Number of connective tissue cells	139,988 ± 1158	134,698 ± 12,922
Numerical density of connective tissue cells	22,653 ± 1188	22,144 ± 2982
Surface area of connective tissue cells (μm^2^)	110 ± 7	102 ± 6
Number of capillary endothelial cells	324,461 ± 35,600 *	296,854 ± 26,658
Numerical density of capillary endothelial cells (mm^−3^)	35,963 ± 4713	37,645 ± 4459
Surface area of capillary endothelial cells (μm^2^)	79 ± 5	75 ± 4
Surface area of glomeruli (μm^2^)	4216 ± 237	4064 ± 535
Distance between glomeruli and Bowman’s capsule	49 ± 3	44 ± 4

**Table 4:** Stereological parameters of the spleen of the control group and treated group of rats; values are shown as mean ± SD; Mann–Whitney *U*-test, * *p* < 0.05.

Parameter	Experimental (*n* = 12)	Control (*n* = 9)
Volume density of epithelial cells (mm^0^)	0.39 ± 0.03	0.36 ± 0.03
Volume density of lymphocytes (mm^0^)	0.42 ± 0.03 *	0.30 ± 0.06
Volume density of connective tissue (mm^0^)	0.13 ± 0.01	0.12 ± 0.03
Volume density of blood capillaries (mm^0^)	0.22 ± 0.03 *	0.17 ± 0.05
Number of epithelial cells	235,889 ± 24,968	204,568 ± 33,318
Numerical density of epithelial cells (mm^−3^)	39,852 ± 3479	35,642 ± 2626
Surface area of epithelial cells (μm^2^)	130 ± 5	132 ± 4
Surface area of nuclei of epithelial cells (μm^2^)	45 ± 7	41 ± 3
NCO of epithelial cells	0.30 ± 0.07	0.29 ± 0.05
Number of connective tissue cells	143,198 ± 8734	137,006 ± 8694
Numerical density of connective tissue cells (mm^−3^)	26,598 ± 3602	23,146 ± 2872
Surface area of connective tissue cells (μm^2^)	113 ± 6	106 ± 6
Number of capillary endothelial cells	249,688 ± 40,834	223,148 ± 20,204
Numerical density of capillary endothelial cells (mm^−3^)	36,426 ± 4879	34,969 ± 2599
Surface area of capillary endothelial cells (μm^2^)	78 ± 4	74 ± 2
Number of lymphocytes	462,635 ± 29,632 *	348,337 ± 23,325
Numerical density of lymphocytes (mm^−3^)	59,226 ± 5059 *	49,132 ± 3740
Surface area of lymphocytes (μm^2^)	95 ± 5	96 ± 5

## Data Availability

The data presented in this study are available in Supplementary Materials.

## Supplementary Materials

The Supplementary Materials are available online at https://www.mdpi.com/article/10.3390/ijms22115468/s1.
