# Supplementary material for: Biocompatibility Study of a New Dental Cement Based on Hydroxyapatite and Calcium Silicates: Focus on Liver, Kidney, and Spleen Tissue Effects
Source: Int J Mol Sci. 2021 May 22;22(11):5468. doi: 10.3390/ijms22115468 (PMC8196841; doi:10.3390/ijms22115468)
Supplement: Supplementary file 1 [file ijms-22-05468-s001.zip › ijms-1196517-supplementary.pdf]

Supplementary Material

1. Brief structural and morphological analysis of ALBO-MPCA

Phase analysis of ALBO-MPCA was performed after 28 days of hydration to investigate the changes inside the initial phases during their curing in water the samples using the X-ray diffraction (XRD) method (Philips PW 1050).

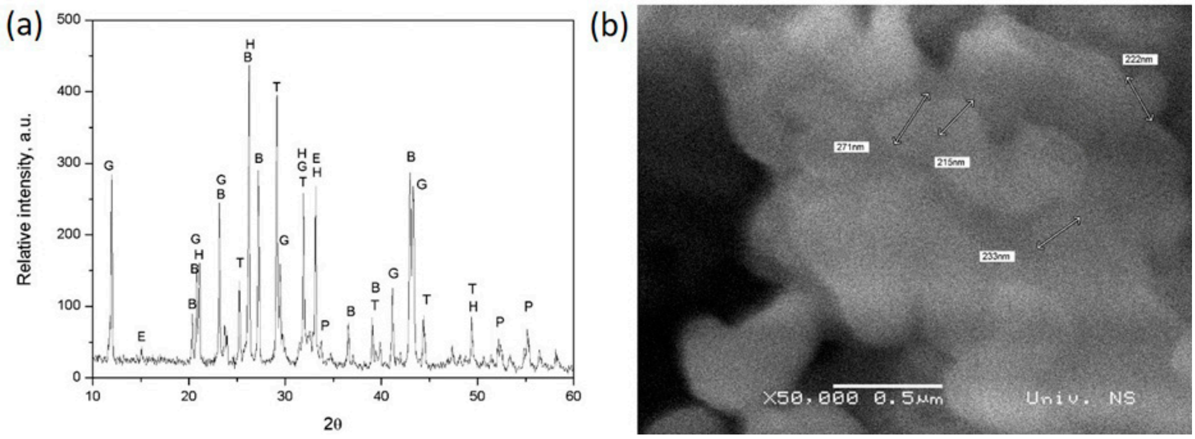

Figure S1. (a) XRD pattern of the ALBO-MPCA cement after 28 days of hydration; (b) SEM micrograph of the ALBO-MPCA cement after hydration.

The XRD pattern (Figure S1a) showed the presence of the following phases: gypsum dihydrate (G) (planes (020), (-121), (031), (-141) and (051) at 2θ of 12.03, 21.13, 23.15, 29.51 and 33.14°, respectively); barite (B) (planes (111), (112), (122) and (312) at 2θ of 23.65, 31.94, 42.94 and 43.35°, respectively) and tobermorite (T) (planes (110) and (112) for 2θ of 29.3 and 29.51°), as prevailing phases. The phases of portlandite (P) (planes (011), (012) and (110) for 2θ of34.86, 48.29 and 50.62°, respectively) and ettringite (E) (planes (110), (304) and (226) for 2θ of 15.17, 33.14 and 39.9°, respectively) were present in small quantities. Hydroxyapatite, as a basic component in the cement, with its characteristic planes (200), (002), (121), (300) and (123) for 2θ

of 21.05, 26.23, 31.87, 33.16 and 49.36°, respectively, was also present. The presence of tobermorite, an important new phase formed during the hydration process, was confirmed, while the absence of starting cement phases β-C_2S and C_3S was also evident.

The morphology of the cement after hydration was analyzed by scanning electron microscopy (SEM) (JEOL, JSM-5300).

Morphology of the cement after 28 days of hydration is shown in Figure S2b. The particles of the cement were packed very close to each other and it it was difficult to precisely determine their particles shape and size. Estimated mean size was between 215 and 270 nm.

The setting time was determined using Vicat needle according to the ASTM C191 standard. The compressive strengths were determined after 24 h, 3, 7 and 28 days of hydration using Instron machine model 4204. The measurements were made on five samples, and the results were given as mean values ± standard deviations. It was found that the setting started after 5±0.1 min and was completed after 15±0.15 min. Compressive strengths were: 32.6±0.5 MPa (24 h), 35.2±0.5 MPa (3 days), 35.8±0.5 MPa (7 days) and 37.4±0.5 MPa (28 days).

2. Detail procedure of animal feeding with material extract/distillated water

The dosage of the extract of cement mixture in concentration 0.1 g/ml was made by using syringe feeding technique. Before the feeding syringe was rinsed with worm water to be sure that is clean. Then by syringe tape was placed in the cement mixture extract, plunger was pulled until all volume of the requested extract was taken. Then, animal was taken by one operator hand. His tomb and forefinger was placed around mouse chest, keeping these fingers under his front legs. After that, the animal was turn towards operator. His right leg was gently turn between operator ring and pinky finger. This action prevent animal from pushing syringe away. Then operator gently turn head towards him. Such position of the mouth is at the angle suitable for easy administration extract from syringe. Then the syringe was placed in mouth, roughly under 450

angle. After the extract was slowly released into the mouth, pausing occasionally to allow the animal to swallow all extract volume. For all animals, during all period this procedure was repeated. By this way, the exact volume of extract was given to all animals, each time.

3. Representative micrographs of histological analysis of liver, kidney, and spleen tissues from control and treatment

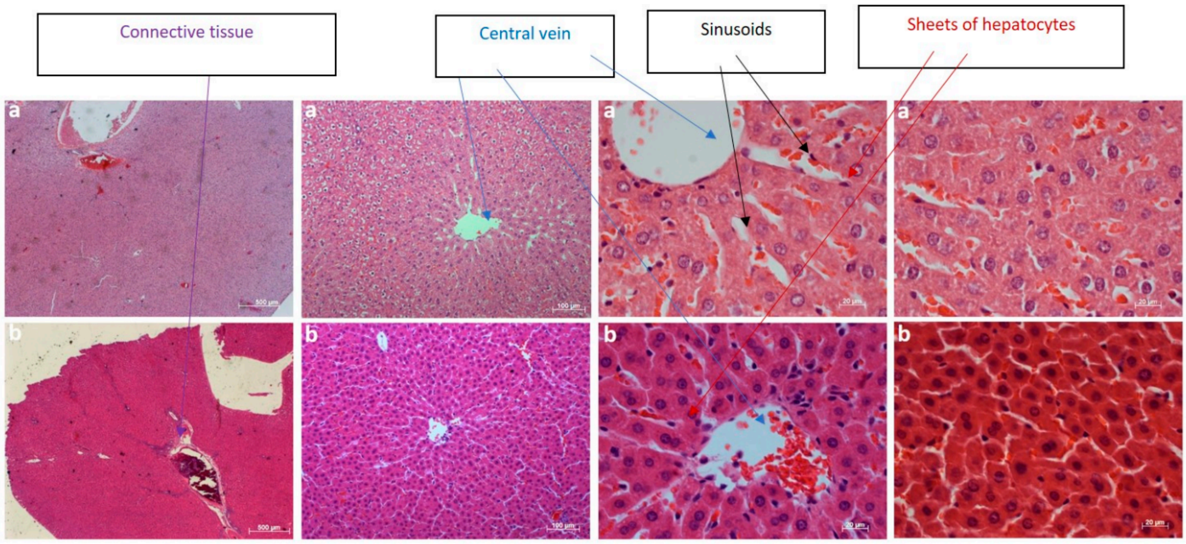

Figure S2. Representative images of histological findings in liver tissue of treatment (a) and control (b) groups after subchronic exposure to ALBO-MPCA, (H&E)

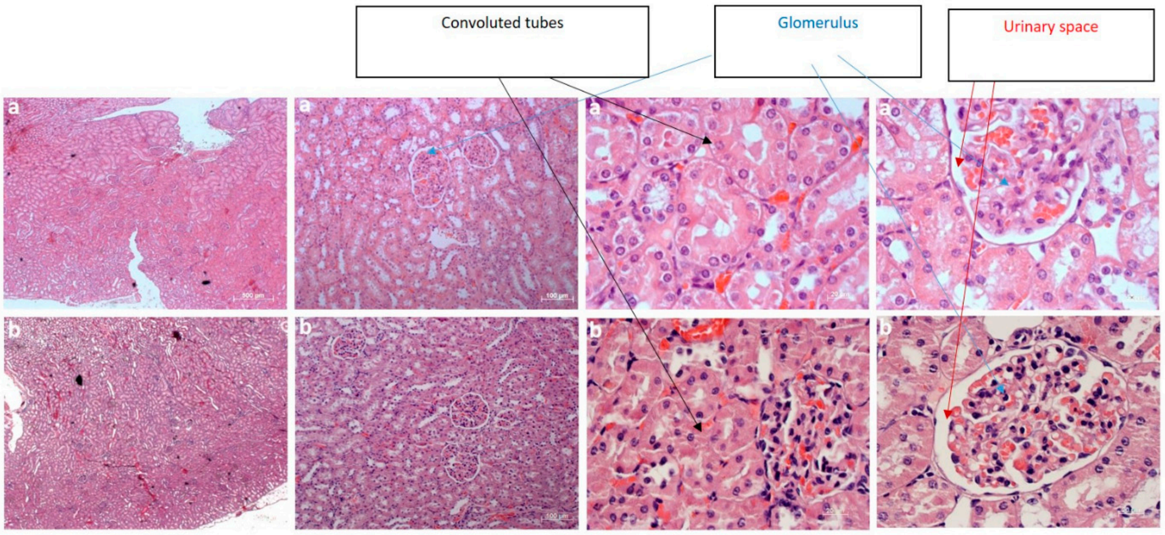

Figure S3. Representative images of histological findings in kidney tissue of treatment (a) and control (b) groups after subchronic exposure to ALBO-MPCA, (H&E)

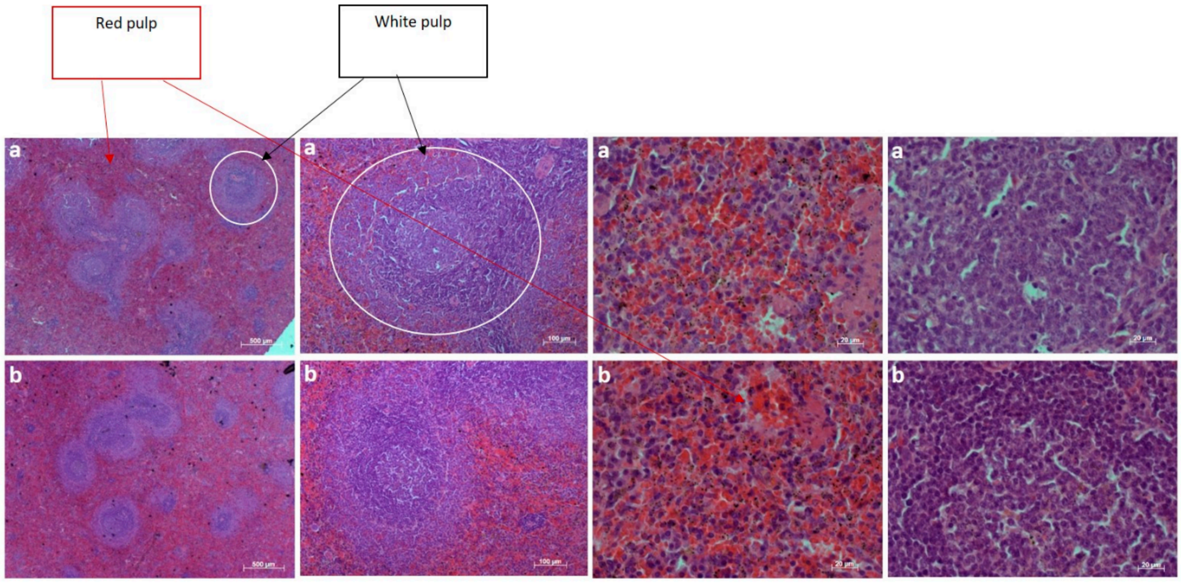

Figure S4. Representative images of histological findings in spleen tissue of treatment (a) and control (b) groups after subchronic exposure to ALBO-MPCA, (H&E)
